# Whether ambulatory electroencephalogram and visual tracking system could be the new strategy for pain assessment?

**DOI:** 10.3389/fnins.2023.1122614

**Published:** 2023-01-17

**Authors:** Xiaqing Ma, Hong Zhang, Tao Xu

**Affiliations:** ^1^Department of Anesthesiology, Suzhou Hospital of Anhui Medical University, Suzhou, Anhui, China; ^2^Department of Anesthesiology, Affiliated Hospital of Nantong University, Medical School of Nantong University, Nantong, China; ^3^Department of Anesthesiology, Shanghai Jiao Tong University Affiliated Sixth People’s Hospital, Shanghai, China; ^4^Department of Anesthesiology, Tongzhou People’s Hospital, Nantong, Jiangsu, China

**Keywords:** pain, assessment, ambulatory electroencephalogram, visual tracking and surveillance system, human

## Abstract

The human pain experience is a complex multi-faceted symptom. Effective pain management begins with a comprehensive assessment. However, a plethora of existing assessment tools for pain assessment focus more on self-report of pain intensity but lack of multi-dimensional impersonal assessment. These unidimensional scales, which capture self-reported levels of pain intensity, not only underestimate the complexity of the pain experience, but also lack stability and objectivity in their own assessments of pain intensity. Therefore, we propose a hypothesis that using scientific and technological means, such as visual tracking and surveillance system, ambulatory electroencephalogram and other techniques, combined with psychological assessment pictures and existing scales, to comprehensively evaluate pain may provide a new method for more effective clinical treatment of pain, especially chronic severe pain.

## Introduction

Pain is defined as “an unpleasant sensory and emotional experience associated with actual or potential tissue damage or described in terms of such damage” by the International Association of the Study of pain. Although acute pain possesses some protective function, when it transforms to chronic, which is defined as persistent or recurring pain that lasts more than 3 months, there is no biological benefit (Treede et al., 2015; Treede, 2019). Therefore, understanding the pathophysiology of pain can improve the treatment and management of pain, which cannot be achieved without a comprehensive assessment of the occurrence of pain.

### Current assessment of pain

The most commonly used assessment tools for pain intensity include the Numerical Rating Scales (NRS), Verbal Rating Scales (VRS), and Visual Analog Scales (VAS) (Hicks et al., 2001; Ferreira-Valente et al., 2011). Participants were told to rate their pain intensity on a scale of 0 (no pain) to 10 (unimaginably severe pain) or given one of several words (no pain, mild pain, moderate pain, severe pain, extreme pain) or more to describe their intensity of pain.

**FIGURE 1:**
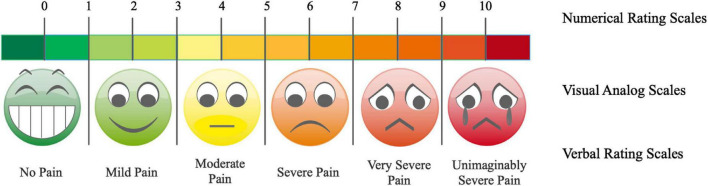
Tradition assessment tools for pain intensity. The Numerical Rating Scales (NRS), Verbal Rating Scales (VRS), and Visual Analog Scales (VAS) are the most commonly used pain intensity assessment methods.

For patients who cannot evaluate the intensity with the above subjective expression, such as the elderly, infants and other groups, bystanders can judge the pain degree of the patients by some pain-related behavioral changes, so as to indirectly evaluate the pain. Under such circumstances, the Faces Pain Scale-Revised (FPS-R) can be used, is represented by six facial expressions (Figure 1), each corresponding to a pain scale of 0–5 (Li and Han, 2019).

There are also many types of pain questionnaires that are more comprehensive than scoring methods elaborated above (Mannion et al., 1996). The most classic and comprehensive is the McGill Pain Questionnaire, which involves not only the intensity of the pain, but also the character of the pain. The questionnaire contains a number of words describing pain that are grouped into four categories: feeling, emotion, evaluation and miscellaneous. The categories were further divided into groups describing different types of pain. In the assessment, the subjects chose the word from each group that best described their pain as closely as possible. However, the questionnaire also has the disadvantages of being unsuitable for people with low educational level, consciousness barrier and communication barrier.

### Shortage of current pain assessments

For acute pain caused by trauma, surgery, etc., determining the location, duration, and intensity of the pain can be of great help in characterizing the pain and evaluating the therapeutic effectiveness of the pain and its underlying cause (Gordon et al., 2016). Whereas, a fundamental acknowledgment is that, to date, no single tool can be widely recommended for assessing acute pain in all contexts.

Unlike acute pain, chronic pain has a significant impact on patients’ physical, emotional and cognitive functioning, social and family life, ability to work and income security (Pereira et al., 2021). Therefore, a comprehensive chronic pain assessment involving the appropriate pain syndrome as well as other perceptual qualities of pain is a more demanding task than assessing acute pain.

Effective relief of dynamic pain is more conductive to postoperative movement, which could reduce the risks of cardiopulmonary and thromboembolic complications, thereby improving long-term outcomes after surgery (Breivik and Stubhaug, 2008; Breivik et al., 2008). So, assessment of the intensity of dynamic pain during mobilization, coughing and deep breathing is more important than acute pain at rest.

For unconscious or sedated intensive care patients, current pain assessment tools have been introduced (Pudas-Tahka et al., 2009; Kerbage et al., 2021). It is important to note that caution should be exercised when using behavioral pain tools because they were developed for patients in one context (e.g., dementia) but may not be appropriate for patients in another context (e.g., patients under sedation in ICU). In addition, the sum of the behavioral pain score differs from the self-reported pain intensity score because it may indicate only the presence of pain but not sensitivity to pain relief (Gordon, 2015).

Clinically, there is another special situation where the assessment of pain in children with poor expression also encounters obstacles. The influence of the environment on pain expression must also be taken into account when assessing children. Healthcare providers and parents may unknowingly prevent children from displaying their pain, and children vary in their ability to develop faked, exaggerated, or suppressed external signs of pain (Von Baeyer and Spagrud, 2007). Therefore, an objective approach is needed to assess pain symptoms in children.

## The hypothesis

We propose a novel concept that utilizing advanced scientific and technological means targeting intra-brain information, such as visual tracking system and ambulatory electroencephalogram with existing scales and questionnaires, to comprehensively evaluate pain may provide a new method for more effective clinical treatment of pain, especially chronic pain.

## Evaluation of the hypothesis

### Visual tracking and surveillance system

Eye tracker is an important instrument in basic research of psychology, which is also widely used in attention, visual perception and reading research to record the characteristics of eye movement when processing visual information (Brigaud et al., 2021).

The structure of modern eye tracker generally includes four systems, namely, optical system, extraction system of pupil center coordinate, superposition system of scene and pupil coordinate and recording and analyzing system of image and data. There are three basic forms of eye movement: fixation, saccades, and pursuit movement (Casas and Chandrasekaran, 2019). According to the research report, the data or parameters commonly used in psychological research using eye tracker mainly include: fixation point trajectory diagram, eye movement time, average speed time and distance in saccadic direction, pupil size, unit pixel and blink (Poletti et al., 2017). The temporal and spatial characteristics of eye movement are the physiological and behavioral manifestations of visual information extraction. It has a direct or indirect relationship with people’s psychological activities. This is why many psychologists devote themselves to the study of eye movement.

The relationship between pain and psychology is complex and multifactorial. The intricate relationship between the two processes has evidenced by the clinical experience. Based on the above introduction of the working principle of eye tracker, we plan to apply it in the clinical diagnosis and treatment of chronic pain. The method is as follows: patients are asked to wear an eye tracker at the first diagnosis and treatment, and the specific characteristics of their chronic pain (including the site of pain, the degree of pain of different sites, emotional impact, etc.) are evaluated comprehensively and objectively. In the subsequent treatment stage, the eye tracker was also worn, and various data were monitored in the background at any time, and the effectiveness of analgesia treatment was evaluated by combining the scale method. We speculate that if eye tracker is applied to pain assessment, it will provide data and analysis beyond the reach of previous means for the assessment of pain intensity and psychological state of pain patients. It will also provide more evidence of its effectiveness in treating patients with chronic pain.

### Ambulatory electroencephalogram

Ambulatory electroencephalography (EEG) system is a portable micro-intelligent recording device. The recorded EEG information can be automatically analyzed by a microcomputer, and the recorded graph and preliminary report can be output by a printer to assist clinical diagnosis (Lawley et al., 2015; Schuele et al., 2021). Laser-evoked EEG responses are increasingly used to investigate nociceptive pathways in fundamental research. The strong repeatable correlation between the intensity of pain perception and the magnitude of the laser-evoked N1, N2, and P2 responses has led some researchers to believe that these responses are directly related to neural activity in the human cortex responsible for encoding pain intensity. Whereas, another research provided compelling evidence to the contrary. They propose that the laser-induced EEG response represents an indirect reading of the function of the nociceptive system. That is, the EEG response is determined not by the perception of pain *per se*, but primarily by the salience of the pain-inducing stimulus (i.e., its ability to capture attention) (Iannetti et al., 2008).

We know that pain, especially chronic pain, is a complex experience, and that aversion to pain can be reflected in the EEG information (Rogenmoser et al., 2021). Therefore, we believe that the greatest advantage of ambulatory EEG combined with pain assessment is that it can combine the timing of pain with the patient’s activity to help effectively control pain.

## Procedure description

**FIGURE 2:**
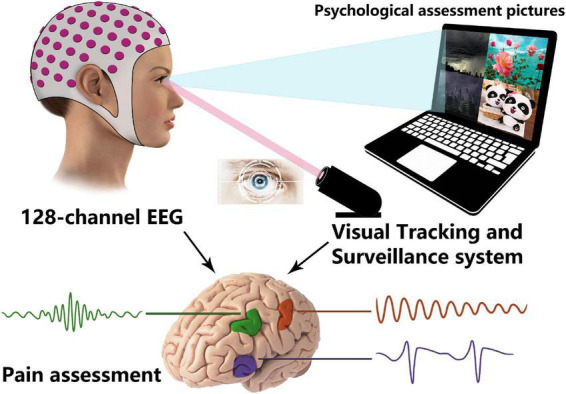
Combination of electroencephalography (EEG) and Visual Tracking and Surveillance System in pain evaluation.

Basing on previous description, integrated mental and psychological behavior, quantifying intra-brain information, and/or combination them with questionnaires might be an effective means to evaluate pain (Figure 2).

As the screen shows different styles’ (positive/negative, comic/tragic) videos and/or pictures (recommended by related psychologists), the visual tracking and surveillance system could be utilized to capture the patient’s visual attention and analysis his/her psychological behaviors. For EEG recordings as biomarkers of pain perception, previous review declared that EEG has potential and future research should be attempted (Panagiotis Zis et al., 2022). Ambulatory EEG could be employed to collect the brain electrical information, which will be used for analysis of the specific characteristics while the patients experience varying degrees of pain. The roles and brain network complexities of the cores also could be calculated and explored basing on these macrodata to identify the universality and complexity of pain.

## Consequences of the hypothesis and discussion

Considering that pain is a complexity syndrome of physical and mental combination, it is far from enough to rely on subjective scores or single evaluated model. By means of current scientific technique, it is feasible to quantify pain from the aspects of mental psychology (visual tracking) and objective processing of information (EEG). Furthermore, existing scales and questionnaires could do an effective supplement. We believe that the combination of visual tracking and surveillance system and ambulatory EEG with existing scales and questionnaires will certainly provide a more comprehensive assessment of pain and thus improve the outcome and quality of patients’ daily life. Even effectively change the current situation of opioid abuse and reverse the opioid crisis in pain control.

## Data availability statement

The original contributions presented in this study are included in this article/supplementary material, further inquiries can be directed to the corresponding author.

## Author contributions

XM and TX designed and conceptualization and revised the final version of the manuscript. XM and HZ drafted the manuscript and prepared the figures. All authors contributed to the article and approved the submitted version.
